# Changes of the Bacterial Abundance and Communities in Shallow Ice Cores from Dunde and Muztagata Glaciers, Western China

**DOI:** 10.3389/fmicb.2016.01716

**Published:** 2016-11-01

**Authors:** Yong Chen, Xiang-Kai Li, Jing Si, Guang-Jian Wu, Li-De Tian, Shu-Rong Xiang

**Affiliations:** ^1^School of Life Science, Lanzhou University, LanzhouChina; ^2^Institute of Modern Physics, Chinese Academy of Sciences, LanzhouChina; ^3^Key Laboratory of Tibetan Environment Changes and Land Surface Processes, Institute of Tibetan Plateau Research, Chinese Academy of Sciences, BeijingChina; ^4^Laboratory of Ice Core and Cold Regions Environment, Cold and Arid Regions Environmental and Engineering Research Institute, Chinese Academy of Science, LanzhouChina

**Keywords:** live cell density, taxonomic group, micro-biogeography, glacier, Tibet Plateau

## Abstract

In this study, six bacterial community structures were analyzed from the Dunde ice core (9.5-m-long) using 16S rRNA gene cloning library technology. Compared to the Muztagata mountain ice core (37-m-long), the Dunde ice core has different dominant community structures, with five genus-related groups *Blastococcus* sp./*Propionibacterium*, *Cryobacterium*-related., *Flavobacterium* sp., *Pedobacter* sp., and *Polaromas* sp. that are frequently found in the six tested ice layers from 1990 to 2000. Live and total microbial density patterns were examined and related to the dynamics of physical-chemical parameters, mineral particle concentrations, and stable isotopic ratios in the precipitations collected from both Muztagata and Dunde ice cores. The Muztagata ice core revealed seasonal response patterns for both live and total cell density, with high cell density occurring in the warming spring and summer months indicated by the proxy value of the stable isotopic ratios. Seasonal analysis of live cell density for the Dunde ice core was not successful due to the limitations of sampling resolution. Both ice cores showed that the cell density peaks were frequently associated with high concentrations of particles. A comparison of microbial communities in the Dunde and Muztagata glaciers showed that similar taxonomic members exist in the related ice cores, but the composition of the prevalent genus-related groups is largely different between the two geographically different glaciers. This indicates that the micro-biogeography associated with geographic differences was mainly influenced by a few dominant taxonomic groups.

## Introduction

A variety of microorganisms including bacteria, archaea, fungi, protozoa, algae, and viruses, and even invertebrates, have been found in glaciers and ice sheets in the Arctic, Antarctic, Greenland, and in other mountains across the world (Skidmore et al., 2005; Nkem et al., 2006; Miteva et al., 2009; Zhang et al., 2009; Branda et al., 2010; Anesio and Laybourn-Parry, 2012; Price and Bay, 2012; Møller et al., 2013; Stibal et al., 2015; Zawierucha et al., 2015; Kaczmarek et al., 2016). Microorganisms can travel long distances and successfully colonize in cryoconite and snow, and then eventually become buried in ice (Prospero et al., 2005; Takeuchi et al., 2006; Miteva et al., 2009; Anesio and Laybourn-Parry, 2012; Yallop et al., 2012; Boetius et al., 2015; Bagshaw et al., 2016). Bacteria are the most dominant life forms in extremely cold, oligotrophic, and frozen water environments. Some of the glacier bacteria have been found to be phylogenetically distinct from those found in temperate environments, demonstrating the biogeography of individual microorganisms in the glacier ice (Christner et al., 2003; Xiang et al., 2010; Anesio and Laybourn-Parry, 2012; Franzetti et al., 2013; Knowlton et al., 2013). Previous studies have also shown apparent geographic patterns of microbial communities across the snow slope surfaces of mountain glaciers Kuytun 51, Qiangyong, and Rongbuk and among the mountain ice cores Dunde (140-m-long, drilled in 1987), Malan (102-m-long, drilled in 1999), Muztagata (37-m-long, drilled in 2003), and Puruogangri (89-m-long, drilled in 2000), and deep ice cores Greenland GISP2D and Antarctic Vostok 5G and Byrd, which illustrates the various microbial responses to climatic and environmental changes of glaciers and ice sheets (Xiang et al., 2009, 2010; An et al., 2010; Knowlton et al., 2013). The micro-biogeography of whole communities may be influenced by the dynamics of taxonomic groups. However, it is still not clear why specific microorganisms live in certain geographical glaciers, namely the geographic difference of the microbial taxonomical groups, which may behave as ecologically coherent units and environmental predictors in glacier systems.

Only a few taxonomic groups are able to colonize and dominate in the snow, although numerous microorganisms are trapped in the surface snow (Zhang et al., 2008, 2009; Xiang et al., 2009, 2010; An et al., 2010). Previous limited data of glacier surface snow have shown that the bacteria *Comamonadaceae* and *Flavisolibacter* sp. are common in both the Kuytun 51 and Qiangyong glaciers but only *Rhodoferax* (*Betaproteobacteria*) is dominant in the Kuytun 51 glacier (Xiang et al., 2010). The changes of the dominant bacteria in glaciers are mainly influenced by processes such as wind deposition (airborne or aerosol-associated microorganisms by prevailing winds and dust-associated microorganisms by dust storm events), precipitation deposition (microbial deposition with snow, wet-deposition), and post-deposition by microbial growth in the warming seasons on the glacier surface snow (Xiang et al., 2009; Price and Bay, 2012; Bottos et al., 2014; Peter et al., 2014; Meola et al., 2015; Miteva et al., 2015; Pearce et al., 2016). Among these processes, post-deposition has an important role in the transition of microbial communities in glaciers. Recent studies have shown influences of post-deposition on the transition of communities from the light-sensitive cyanobacteria dominated in the surface snow to the non-light-sensitive bacteria buried in the subsurface snow (Xiang et al., 2009). The geographic differences in microbial communities across the mountain glaciers could be attributed to the mountain barriers, which might control the microbial deposition by changing the prevailing wind directions and moisture sources; while the geographic patterns of the dominant microbial colonizers in glaciers might be also influenced by the local climatic and environmental conditions (Nkem et al., 2006; Xiang et al., 2009, 2010; Demetras et al., 2010; Meola et al., 2015).

The primary goal of this study was to evaluate how the geographic difference of bacterial communities at a taxonomic group level was controlled by the prevailing wind patterns across the mountain glaciers in western China. We investigated two different glaciers, the Muztagata glacier (38°17′N, 75°04′E) and the Dunde ice cap (38°06′N, 96°24′E). Six structures of bacterial communities were established from the Dunde ice core columns (at field depth 0.8–5.3 m) using bacterial 16S rRNA gene clone library technology. Additionally, live bacteria were examined and related to the physical-chemical parameters from the Muztagata and Dunde ice cores.

## Study Area, Data Collection, and Methodology

**FIGURE 1:**
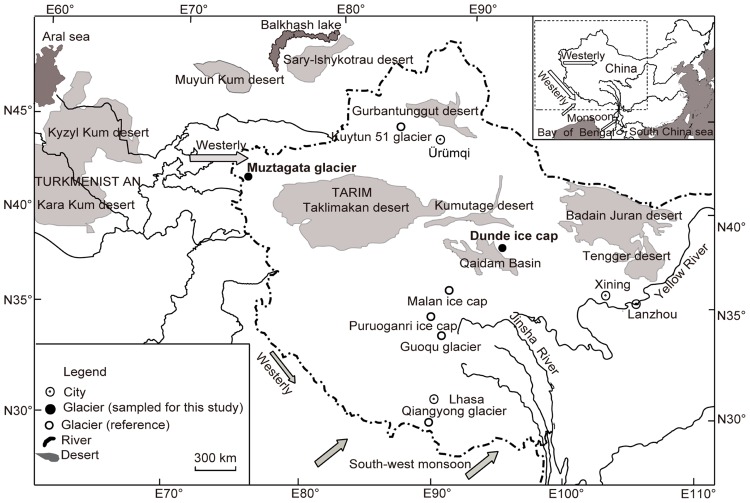
**Map illustrating the location of glaciers discussed in this study**.

In this study, data were collected from the Muztagata Glacier (38°17′N, 75°04′E), the Dunde ice cap (38°06′N, 96°24′E), and the Puruogangri ice cap (33°54′N, 89°10′E) where precipitation patterns were mainly controlled by two different circulations- westerly and monsoon (as indicated by the highlighted arrows in the **Figure 1**; **Table 1**). The Muztagata Glacier is located in the most western margin of the Tibetan Plateau, where precipitation is mainly controlled by westerly circulation originating in the arid and semi-arid regions, including the deserts Sary-Ishykotrau, Muyun Kum, Kyzyl Kum, Kara Kum, Taklimakan, and Gurbantunggut (Wake et al., 1990). The Dunde ice cap is located in the northern margin of the Qaidam Basin and in the Qilian mountain region on the northeastern Tibetan Plateau, where the winter precipitation results from the incursion of westerly depressions along the southern slopes of the Himalaya (Murakami, 1987; Davis et al., 2005), while the summer precipitation is derived from the monsoon circulation from the Bay of Bengal to central Himalaya, and further to the Qaidam Basin and large depressions in Takalimakan Desert and Daidam Basin (Chen and Bowler, 1986; Davis et al., 2005). The Puruogangri ice caps are located in the center of the Tibetan Plateau, where precipitation is derived from a westerly direction during winter and Indian monsoons in the summer (Wake et al., 1993; Shi and Liu, 2000).

**Table 1:** Ice core location and characteristics.

Ice core	Position	Elevation (m)	Length (m)	Age of core (Calendar year)	Date drilled	Moisture sources		Reference
						Summer	Winter	
Muztagata	38°17′N, 75°04′E	7010	37	1963–2000	Summer 2003	Westerly	Westerly, Polar air masses	This study; Wake et al., 1990; Tian et al., 2006; Yu et al., 2006
Dunde	38°06′N, 96°24′E	5325	9.5	1980s–2000	Oct, 2002	Monsoon	Westerly	This study; Chen and Bowler, 1986; Murakami, 1987; Davis et al., 2005
Puruoganri	33°54′N, 89°10′E	6200	89	1600s–2000	Oct, 2000	Monsoon	Westerly	Wake et al., 1993; Shi and Liu, 2000; Zhang et al., 2009

The ice core Muztagata (37-m-long) was extracted at 7010 m ASL (above sea level) from the Muztagata Glacier in the summer of 2003 (Tian et al., 2006). The Dunde ice core (9.5-m-long) was extracted at 5325 m ASL from the Dunde ice cap summit in October 2002 (Wu et al., 2009). The visible stratigraphic features were recorded immediately after ice core drilling. All ice cores were returned frozen to the freezer room (air temperature between -18°C to -24°C) at the Key Laboratory of the Ice Core and Cold Regions Environment of the Chinese Academy of Sciences. The ice core sections were split lengthwise into four portions and stored in a refrigerated room with a temperature of -18°C to -24°C.

A 10 ml aliquot of melt-water from the Muztagata and Dunde ice cores was used for the analysis of the mineral particles. Total micro-particle concentrations were measured by using a Coulter counter Multisizer 3 (Beckman). A total of 44 ice samples were analyzed from the Muztagata ice core taken at a depth of 2.5–12.5 m, and 74 samples were analyzed from the Dunde ice core taken at a depth of 0.50–9.8 m.

A 10 ml aliquot of melt-water from the Dunde ice core was used for analysis of the stable isotopic ratios, ^18^O/^16^O (δ^18^O) in the precipitation. A Finnegan MAT-252 mass-spectrometer was used to determine δ^18^O values within ± 0.05%. The Dunde ice core was dated by using seasonal δ^18^O variations and annual visible dust layers and confirmed by the previous data (Takeuchi et al., 2009). The Muztagata ice core dating and δ^18^O data were previously described by Tian et al. (2006).

The ice core sections were cut into small ice columns in intervals of 12–30 cm using a band saw within the walk-in freezers (-18°C to -24°C). Microbial analyses were carried out on 156 and 37 samples from Muztagata and Dunde, respectively. The ice samples were cut between the visible dust layers, and ice layers were collected separately. The improved procedures were used for the decontamination of the outer surfaces of ice core samples. The snow and firn-ice columns (length approximately 15 cm, diameter 5 cm) were decontaminated by cutting away the 10-mm annulus with an autoclaved sterile sawtooth knife. The knife was sterilized over an alcohol flame following each ice slice cut. A total of three sterile sawtooth knives were used for each ice sample. The decontaminated samples were then completely melted in clean and sterile glass beakers at 4°C. These handling procedures were undertaken at temperatures below 20°C within a sterile, positive pressure laminar flow hood as described before (Yao et al., 2006). The freshly melted water (10 ml) from the Muztagata and Dunde ice cores was 10-fold diluted with sterile filtered water. A total of 100 μl of diluted sample was added to the known concentration of fluorescent-dyed bead solution Trucount (Becton Dickinson) mixture with the cell sorting markers carboxyfluorescein diacetate (cFDA) and propidium iodide (PI). Three groups of bacteria could be identified based on the difference of the bound probes: cFDA-stained, cFDA/PI-double-stained, and PI-stained group, indicating viable, injured, and dead cells, respectively (Xiang et al., 2009). The cFDA and PI staining were separately prepared by following the method of Amor et al. (2002), except for the cell suspensions that were incubated for 15 min in the dark at room temperature (25°C) for cell staining. The 100 μl sterile filtered water served as a reagent blank. The live and total cell numbers in the melt-water were determined with a precision ± 0.05% by using a FACSCalibur flow cytometer (Becton Dickinson Immunocytometry Systems, San Jose, CA, USA) and following the manufacturer’s instruction.

For DNA analysis, six clone libraries of the bacterial 16S rRNA genes were collected from the Dunde ice cap. Approximately 400 ml of ice core melt-water was used for the DNA extraction. DNA extraction and further clone library establishment procedures were conducted by following the same protocols as previously used in a microbial analysis of the Kuytun 51 Glacier samples (Xiang et al., 2009). All reagent transfers for DNA analysis were performed within a sterile, positive pressure laminar flow hood. All reaction tubes and micropipette tips were autoclaved, and all solutions except for the Taq DNA (2.5 U, TakaRa) polymerase were passed through sterile 0.2 μm filters (Xiang et al., 2004). The 16S rRNA gene amplicons used for the establishment of clone libraries from the Dunde ice core were generated by PCR amplification with the bacterial universal primer pair 8f (5′-AGAGTTTGATCATGGCTCAG) and 1492R (5’-CGGTTACCTTGTTACGACTT; Lane, 1991; Weisenburg et al., 1991). To avoid possible bias, the three PCR products were pooled and used to establish a clone library from each ice column. A total of 137 clones were selected for sequencing by *Hae*III-based ARDRA (amplified rRNA restriction analysis) out of the 406 clones from the Dunde ice core. Each sequence was named using the initial of Dunde ice cap (DD1, noted for one out of the five ice cores drilled in October 2002, Wu et al., 2009), along with the ice depth (D84, D107, D238, D324, D386, and D466: 84, 107, 238, 324, 386, and 466 cm below the snow surface) followed by the clone number (1–163). For example, clones DD1D84-9, DD1D107-55, and DD1D466-123 were the clone representatives of the ice core DD1 taken at the depth 84, 107, and 466 cm below the snow surface. The GenBank accession numbers of the cloned sequences obtained from the Dunde ice core are KU060881–KU061017.

All 137 sequences from the Dunde ice cap were checked by DECIPHER (Wright et al., 2012, sequence chimera check tool^1^) and aligned with the Blast references (Altschul et al., 1990) by using ClustalX (Thompson et al., 1997). A Neighbor-Joining phylogeny for the aligned sequences was constructed using MEGA 6.0^2^ (Tamura et al., 2013) pairwise deletion mode for gaps (with bootstrap analysis, 100 replicates) and subroutines Maximum Composite Likelihood (MCL) for substitutions. The archaeal 16S rDNA sequences from *Methanosaeta harundinacea* strain 8Ac (accession no. AY817738) and *Methanosaeta concilii* strain GP6 (accession no. NR102903) were used as outgroup references on all trees. All the obtained sequences from the glaciers were identified by the recognized species and were related to the ecological clusters (e.g., *Variovorax* sp. and *Herbaspirillum* sp. in the *Betaproteobacteria* subphyla). Sequences obtained displaying similarities of >97% with known species were identified as the reported species. Most of the obtained clones were related to known cultivated genera or genus clones (e.g., *Ketogulonicigenium* sp., *Cyanobacterium* sp., and *Sphingobacterium* sp.). A few clones had <97% similarity with reported species, and thus were designated separately.

## Results

### Seasonal Changes in Physical-Chemical and Biological Parameters in the Muztagata Ice Core

**FIGURE 2:**
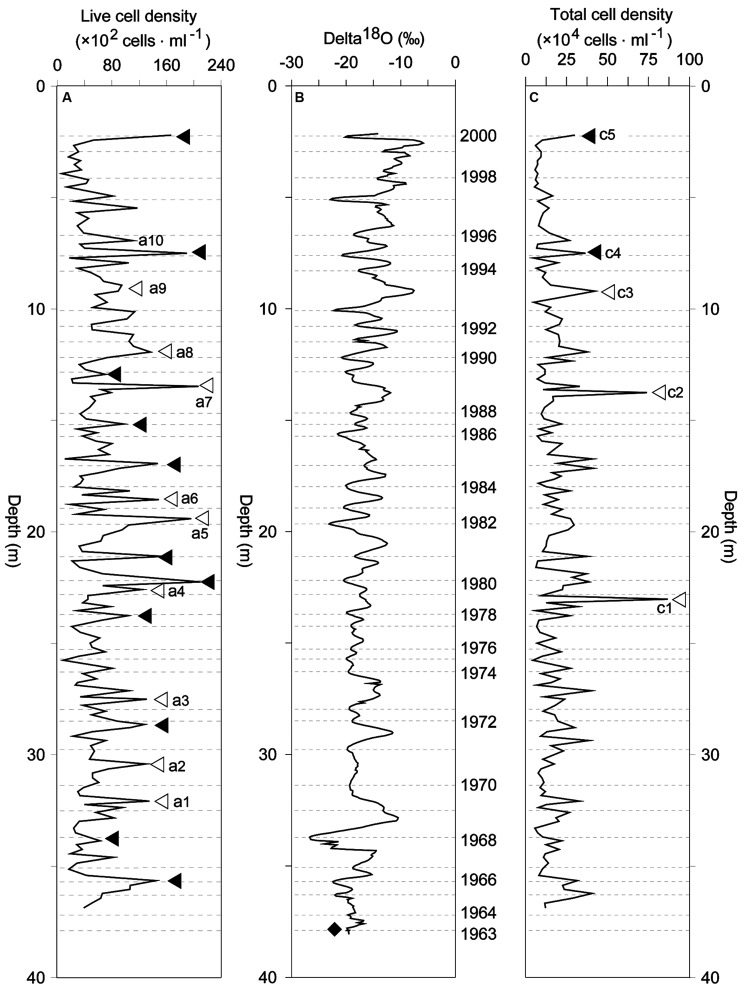
**Bacterial cell density and δ^18^O in the Muztagata ice core.** The Muztagata ice core (37-m-long) was extracted at 7010 m ASL from the Muztagata Glacier in the summer of 2003. **(A)** Live cell density in the ice. **(B)** The δ^18^O value was measured by Finnegan MAT-252 mass-spectrometer (adapted from Tian et al., 2006). Ice core was annually dated by using seasonal δ^18^O variations and annual visible dust layers, and the peak of beta radioactivity by the nuclear weapon test in 1963 was identified at a depth of 37.89 m (Tian et al., 2006). **(C)** Total bacterial cell density estimated by using flow cytometer and cFDA/PI-stain, see the detailed in the “Study Area, Data Collection, and Methodology”). The annual ice layers ranged from 50 to 136 cm, and the years were indicated by the dash lines in the **(A–C)**. The data presented here were only for the ice core section in a depth range from 2.21 to 37 m since the annual layers become thinner below 35 m and the ice layer being near the bottom of the glacier (the depth of the glacier is 52.6 m, Tian et al., 2006).

**FIGURE 3:**
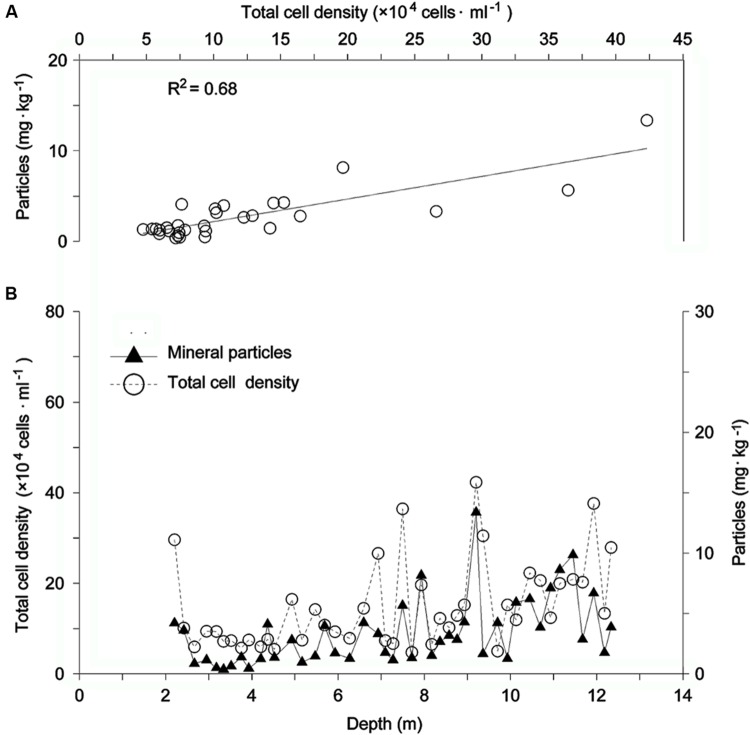
**Correlation between mineral particle concentrations and total cell density in the Muztagata ice core. (A)** Correlation between total cell density and mineral particle concentrations at ice core depth 2.5–9.3 m. **(B)** Total bacterial cell density and mineral particle concentrations. Total microparticle concentrations were measured by using a Coulter counter Multisizer3 (Beckman).

There was an obvious seasonal effect on temperature and biological parameters along the ice core extracted at 7010 m ASL of the Muztagata Glacier (**Figure 2**). An apparent seasonal temperature change was indicated by the proxy value of the stable isotopic ratios, ^18^O/^16^O (δ^18^O), with a low value in winter and a high value in summer (**Figure 2B**). The live cell density was greatly variable and ranged from 6.5 × 10^2^ to 2.1 × 10^4^ cells/ml between 1964 and 2000 (**Figure 2A**). The total cell density varied from 4.4 × 10^4^ to 8.7 × 10^5^ cells/ml (**Figure 2C**). Several live cell density peaks were formed during the summer seasons in 1969, 1970, 1973, 1979, 1982, 1983, 1988, 1990, and 1993 for a total of nine events, a1 to a9 (open triangles in **Figure 2A**), while cell density peaks were found in spring (filled triangles in **Figure 2A**). This ice core also had an increased density of the total number of microorganisms in the summer of 1978, 1988, and 1993 (open triangles c1, c2, and c3 in **Figure 2C**), and in the spring of 1995 and 2000 (c4 and c5 in **Figure 2C**), which was consistent with the live cell density patterns (**Figure 2A**). The microbial cell density correlated with the concentrations of mineral particles and possessed a high *R*^2^ value of 0.68 only from 1994 to 2000 (from ice core depth 2.5 to 9.3 m, **Figures 3A,B**), but did not correlate with mineral particle concentrations from 1990 to 1993 (from ice core depth 9.3 to 12.5 m, with *R*^2^ < 0.1, **Figure 3B**).

### Changes in Physical-Chemical and Biological Parameters in the Dunde Ice Core

**FIGURE 4:**
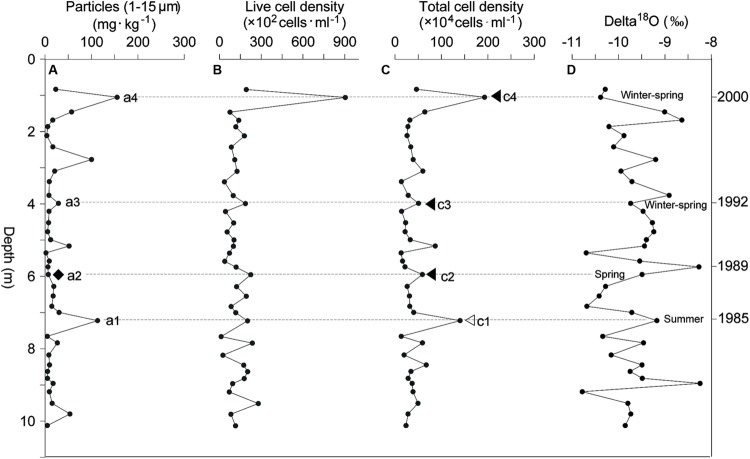
**Bacterial cell density, mineral particles, and δ^18^O in the Dunde ice core. (A)** Mineral particle concentration along the depth profile. Total microparticle concentrations were measured by using a Coulter counter Multisizer3 (Beckman). **(B)** Live cell density. **(C)** Total bacterial cell density. The live and total bacterial cell density were estimated by using flow cytometer and cFDA/PI-stain. **(D)** δ^18^O value. The δ^18^O value was measured by Finnegan MAT-252 gas stable isotope ratio mass-spectrometer. In this study, the ice core section at 0.54–9.81 m depth was dated by using seasonal δ^18^O variations and annual visible dust layers, and confirmed by the 51-m-long Dunde ice core drilled at the same site and in the same year 2002 (Takeuchi et al., 2009). The winter-spring seasons of 1992 and 2000 were identified and confirmed by the deep valleys of δ^18^O and dust peaks, the summer of 1989 was identified by the peak of δ^18^O, and the summer of 1985 was confirmed by both of the δ^18^O and dust peaks, respectively.

Seasonal analysis of the Dunde ice core was not successful due to the limitations of sample resolution (**Figure 4**). Oxygen isotope ratios of the melt-water samples from the Dunde ice core showed a change range from –10.78‰ to –8.24‰ (temperature proxy ^18^O/^16^O, **Figure 4D**), while microbial cell density varied from 1.2 × 10^3^ to 9.1 × 10^4^ cells/ml (**Figure 4B**) and 1.3 × 10^5^ to 1.9 × 10^6^ cells/ml (**Figure 4C**) for live and total cell density, respectively. Three peaks c2, c3, and c4 of the total cell density were found in the spring of 1988–1989, 1992, and 2000, only one peak, c1, was found in the summer of 1985 (**Figure 4C**). The live cell density response pattern was consistent with the total cell density tendency (the dash lines in **Figures 4B,C**). An abundance of microbial cells frequently occurred at the dirty ice layers (Cell density peaks c1, c3, and c4 at the dust layers labeled as a1, a3, and a4 at the dash lines in **Figures 4A,C**), but were rarely found at the clean ice layer (small density peak c2 at the a1 ice layer in **Figure 4**).

### Phylogenetic Analysis of Bacterial 16S rRNA Gene Amplified from the Dunde Ice Core

**FIGURE 5:**
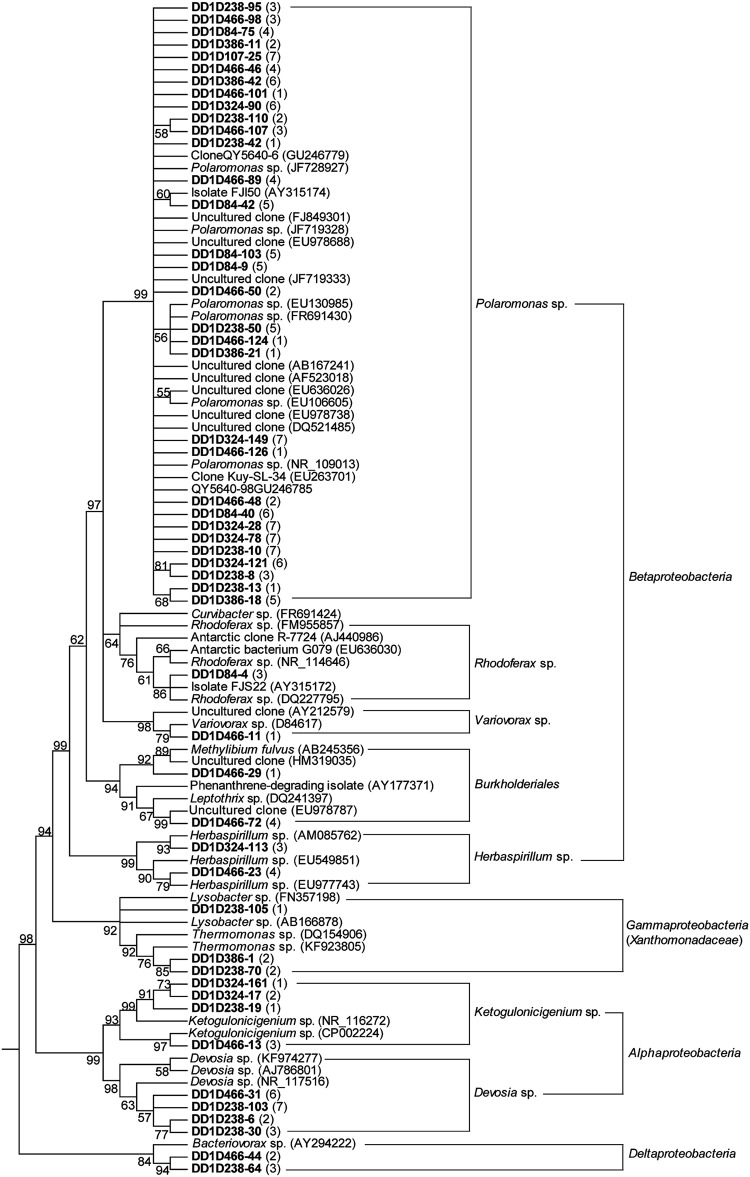
**Phylogenetic analysis of the 16S rRNA genes for *Alphaproteobacteria*, *Betaproteobacteria, Gammaproteobacteria*, and *Deltaproteobacteria* clones from the Dunde ice core and the closest relatives.** The tree was generated by the Neighbor-Joining method after sequence alignment, and rooted with two *Methanosaeta* strains (accession no. AY817738 and NR102903). Bootstrap values (100 replications) were specified for each Node. Cut-off value for the condensed tree was 55%. Numbers of the obtained snow-ice clones (had the same ARDRA pattern to the sequenced representatives listed on the tree) and relative sequence affiliations corresponding to GenBank accession number were provided in parentheses. The sequences discussed in this study were noted bold. See a detailed description for the assigned sequence references and numbers in “Study Area, Data Collection, and Methodology.”

**FIGURE 6:**
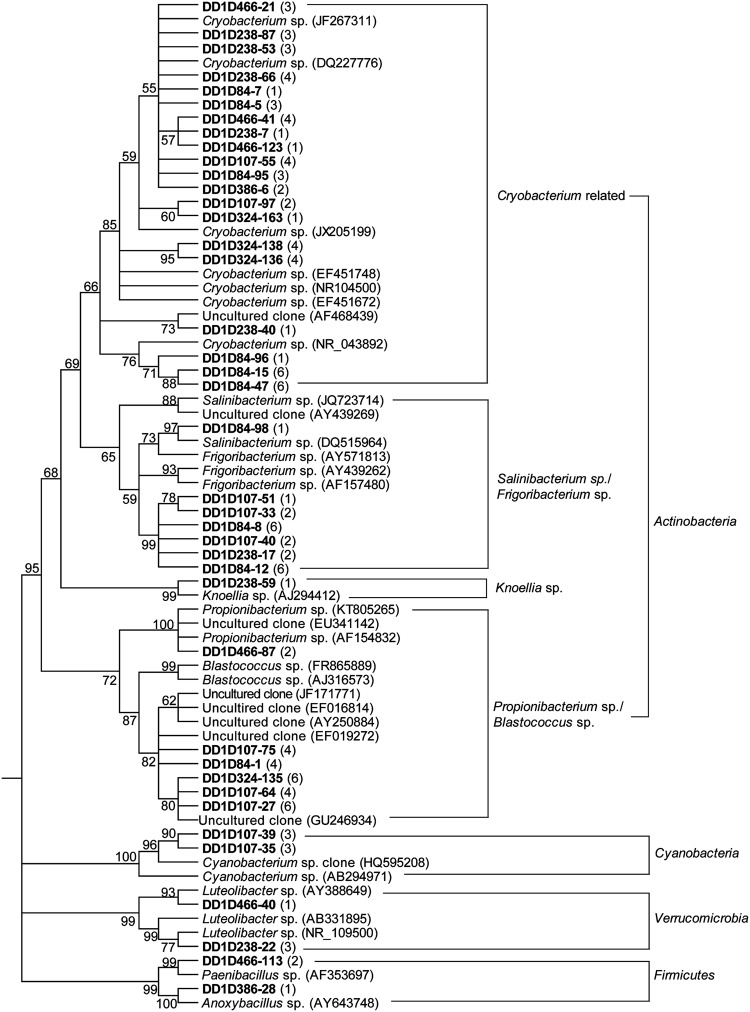
**Phylogenetic analysis of the 16S rRNA genes for the *Actinobacteria*, *Cyanobacteria*, *Verrucomicrobia*, and *Firmicutes* clones from the Dunde ice core and the closest relatives.** The tree was constructed by following the protocol as described in **Figure 5**.

**FIGURE 7:**
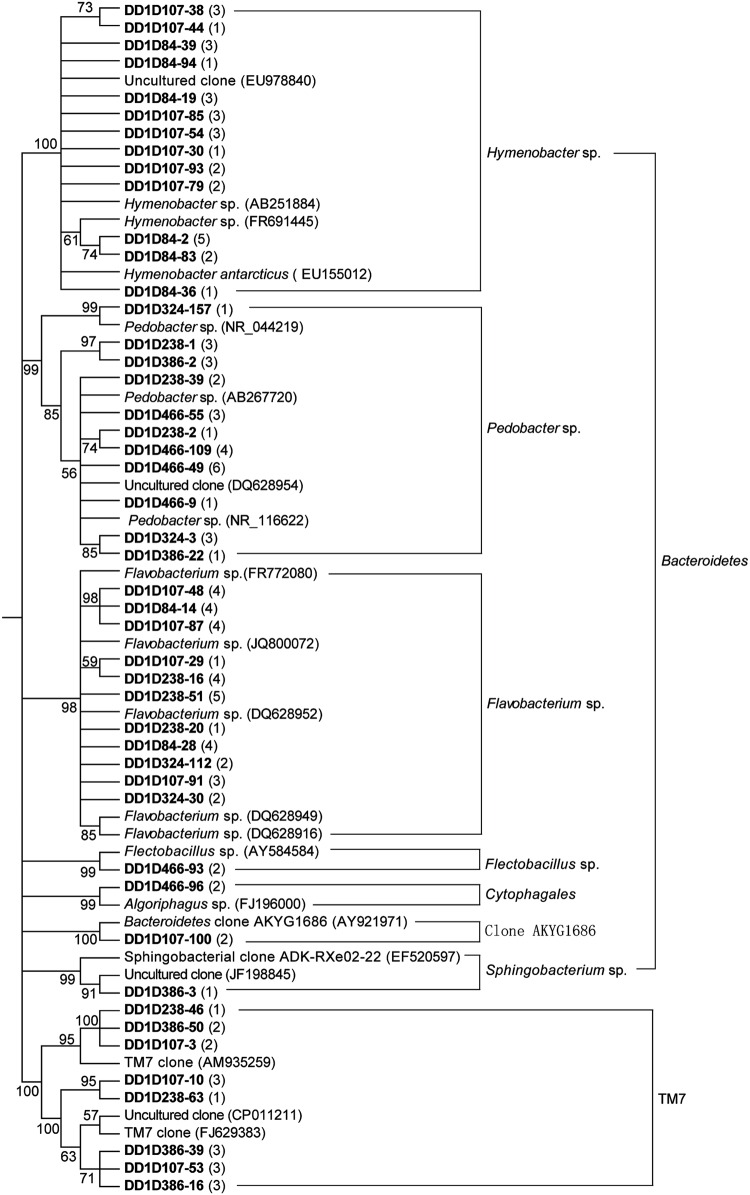
**Phylogenetic analysis of the 16S rRNA genes for the *Bacteroidetes* and TM7 candidate clones from the Dunde ice core and the closest relatives.** The tree was constructed by following the protocol as described in **Figure 5**.

The dominant bacteria in six ice layers of the Dunde ice core were investigated using 16S rRNA gene clone library, sequencing techniques, BLAST and phylogenetic tools. A total of 24 bacterial genera were identified in the Dunde ice core. They belonged to genera *Polaromonas* sp., *Rhodoferax* sp., *Variovorax* sp., *Burkholderiales*, *Herbaspirillum* sp., *Xanthomonadaceae*, *Ketogulonicigenium* sp., *Devosia* sp., *Bacteriovorax* sp., *Hymenobacter* sp., *Pedobacter* sp., *Flavobacterium* sp., *Flectobacillus* sp., *Cytophagales*, *Sphingobacteriaceae*, *Cryobacterium*-related, *Propionibacterium*/*Blastococcus* sp., *Salinibacterium*/*Frigoribacterium* sp., *Knoellia* sp., *Cyanobacteria*, *Luteolibacter* sp., *Paenibacillus* sp., *Anoxybacillus* sp., and TM7 candidates (**Figures 5–7**). Three genus groups *Cryobacterium*-related, *Salinibacterium*/*Frigoribacterium* sp., and *Propionibacterium*/*Blastococcus* sp. were clustered with 65–76% similarity to the known species but grouped with genus *Knoellia* sp. with 95% similarity in the family members of *Actinobacteria* (**Figure 6**). Only one clone DD1D107-100 was 100% similar to the uncultured *Bacteroidetes* clone AKYG1686 (**Figure 7**). All tested bacterial clones in the ice fell into members of bacteria phyla *Alpha*, *Beta, Gamma, and Deltaproteobacteria*, *Actinobacteria*, *Bacteroidetes*, *Firmicutes, Verrucomicrobia*, and TM7 candidates.

### Changes in Proportion of the Main Bacterial Genera along the Dunde Ice Core Profile

**FIGURE 8:**
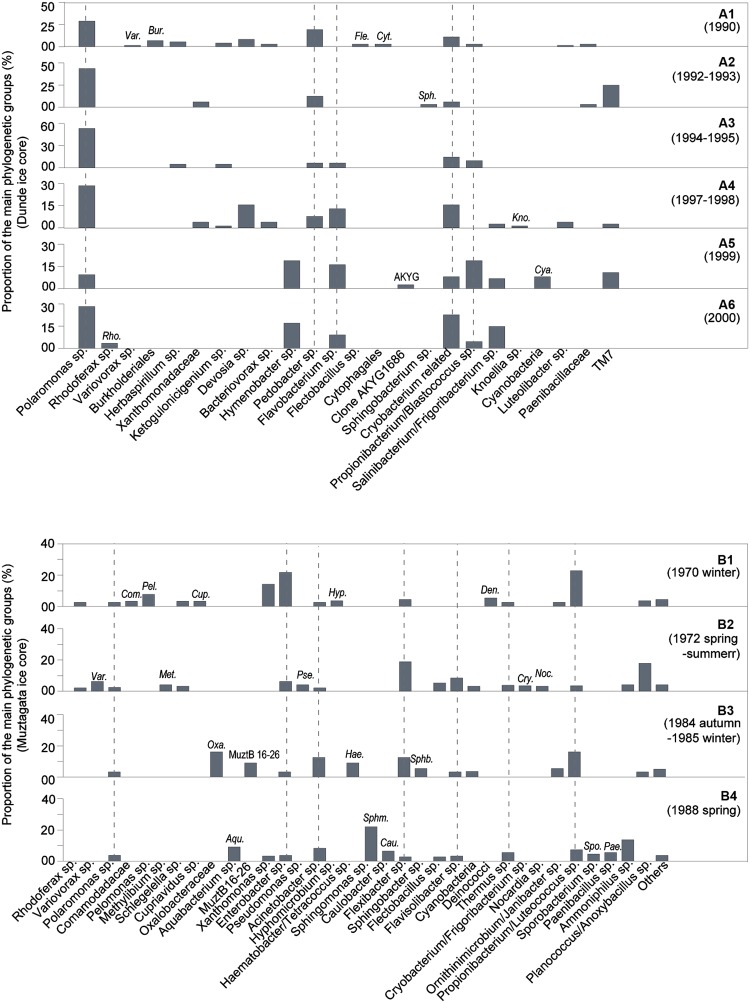
**Proportion of the main phylogenetic groups in the Dunde and Muztagata ice cores. (A1–A6)** Dominant bacteria in six Dunde ice columns dated in the calendar year 1990, 1992–1993, 1994–1995, 1997–1998, 1999, and 2000, respectively. **(B1–B4)** Dominant bacteria in four Muztagata ice columns dated in 1970, 1972, 1984–1985, and 1988, respectively. Muztagata ice core dada was adapted from our previous report (An et al., 2010).

There was a large difference in the proportion of the main phylogenetic groups along the Dunde glacier depth profile, which indicated the seasonal changes of microbial communities in the glacier (**Figures 8A1–A6**). The bacterial clones were comprised of five dominant genus groups, *Polaromonas* sp., *Pedobacter* sp., *Flavobacterium* sp., *Propionibacterium*/*Blastococcus* sp., and *Cryobacterium*-related, which accounted for more than 55% of the total 406 clones and frequently appeared in the six tested ice layers from 1990 to 2000 (dashed lines in **Figures 8A1–A6**). Nine genus groups such as *Rhodoferax* sp., *Variovorax* sp., *Burkholderiales*, *Flectobacillus* sp., *Cytophagales*, *Sphingobacteriaceae*, *Knoellia* sp., *Cyanobacteria* rarely occurred in the ice. Other opportunistic bacterial clones occasionally appeared in the ice.

## Discussion

Previous studies have shown the prevalence of specific bacteria in certain local glaciers (Zhang et al., 2008; Xiang et al., 2009; An et al., 2010; Franzetti et al., 2013; Miteva et al., 2015). However, our findings demonstrate that the members of bacterial genus-related groups are highly similar in the related ice cores at a historical scale, whereas the composition of the prevalent genus-related groups is largely different across the geographically different glaciers. This indicates that the micro-biogeography associated with geographic differences was mainly influenced by a few dominant taxonomic groups.

### Methodological Considerations

Contamination of the DNA samples (from the inner core columns) used in this study is unlikely because the outer surfaces of ice core and reagents for DNA analysis were cautiously decontaminated, and all the procedures were performed within a sterile, positive pressure laminar flow hood. Only small DNA fragments (<100 bp) were detected from the ice column control of (autoclaved sterile water), which were not considered for further sequence analysis in this study. It should be noted that *Herbaspirillum* sequences, also found in this study, have previously been identified as potential contaminants in glacier debris and ice samples (Cameron et al., 2016). However, the experimental procedures used by Cameron were completely different from ours in the present study. Their protocols and procedures were used for glacier cryoconite debris and surface ice samples. *Herbaspirillum* sp. found in this study, are well-known plant root-associated nitrogen-fixing (Baldani et al., 1986) and non-nitrogen-fixing environmental species (Ding and Yokota, 2004; Dobritsa et al., 2010). They were also reported in the Alaska Gulkana glacier, an Antarctica glacier forefield and the Antarctica Lake Vida brine (Segawa et al., 2011; Bajerski et al., 2013; Kuhn et al., 2014).

Various molecular techniques, CFM with cell stains cFDA, PI, and SYTOX have been used to investigate viable bacteria (Amor et al., 2002; Schumann et al., 2003; Xiang et al., 2009). These tools helped us to examine the abundance of live cells and the potential metabolic activities of microorganisms in an environment. However, the CFM approach has certain limitations because of interference from dust particles or spurious abiotic autofluorescence and underestimation of the accurate cell counts under the typical CFM parameters (Stibal et al., 2015). Despite the limitations, the background noise can be counterweighed by data series from the ice core profiles. In this study, the apparent seasonal tendency suggests that our analyses were based on a substantial fraction of bacteria.

For the phylogenetic analysis of bacteria, more than 600 clones were picked and sequenced. A total of 406 valid bacterial clones were obtained from the Dunde ice core after vector, and chimeric checking. The rarefaction curves of six clone libraries from the ice core were approaching asymptotes (dada not shown). Data also showed the prevalence of a few dominant genus-related groups at the different ice core depths (**Figures 8A1–A6**). This indicated that the identified clones were based on the dominant bacterial taxa.

### Dust Deposition and Microbial Distribution along the Glacial Depth Profiles

The present data sets from the Muztagata glacier at 7010 m ASL (38°17′N, 75°04′E) revealed a high correlation between dust and microbial abundance from 1994 to 2000, which indicated a strong influence of aeolian activities on the microbial deposition in the glacier snow (**Figures 3A,B**). This was also consistent with another independent microbial investigation on the Muztagata glacier at 6300 m above sea level (Liu et al., 2013). The Dunde ice core also presented a frequent association of microbial cell density peaks with high concentrations of mineral particles (a1, a3, and a4, verse c1, c3, and c4 in **Figures 4A,C**). The strong association of microorganisms with dust was also found in previous data from the Antarctic glacier (Abyzov et al., 1998; Priscu et al., 2008), the Malan glacier (Yao et al., 2006), and the Guoqu glacier on the Tibetan Plateau (Yao et al., 2008). The analyses of trace and rare earth elements extracted from the same series of Dunde ice core sections showed that the fine fractions in the Dunde dust were more similar to those in the western Qaidam Basin and Tarim Taklimakan Desert than those in the Badain Juran and Tengger Desert (Wu et al., 2009). The Nd-Sr isotopic composition of mineral particles in the Dunde ice core is also similar to that in desert sand from Qaidam and Tarim Taklimakan (Wu et al., 2010). All results revealed that the Qaidam Basin and the Tarim Taklimakan Desert was the main source of dust in the Dunder glacier, implying the transportation of dust-borne microorganisms from the western desert Tarim Taklimakan and adjacent Qaidam to the Dunde glacier.

However, the Muztagata ice core data showed independence of microbial load with dust deposition from ice core depth 9.3 to 12.5 m (**Figure 3B**). The Dunde ice core data also showed one small cell density peak c2 appearing at the clean ice layer a2 in **Figure 4**. These results indicate that microbial deposition in the glacier snow does not always associate with the dust deposits or “dirty” wind and may in fact be transported by “clean” wind or snow, which implies influences of the processes such as aerosol and precipitation deposition, along with other factors (Bottos et al., 2014; Pearce et al., 2016).

### Seasonal Fluctuation of Bacterial Density at Variable Temperatures

The present data sets from the Muztagata glacier at 7010 m ASL (38°17′N, 75°04′E), revealed clear seasonal patterns with high microbial cell density occurring in the warming summer months (open triangles **Figure 2**), which indicated positive temperature effects on the microbial density patterns. This was consistent with another independent microbial investigation on the Muztagata glacier at 6300 m ASL (38°17′N, 75°06′E, Liu et al., 2013). The high repeatability of both ice cores from the Muztagata glacier confirmed the reliability of the data sets discussed here. Evidence for a positive temperature effect includes the algae growth of red *Chlamydomonas* at the surface snow in New Zealand and on the Alaska Harding icefield and on Greenland and Iceland’s glaciers in late spring and summer (Thomas and Broady, 1997; Takeuchi et al., 2006; Yallop et al., 2012; Lutz et al., 2015). Further temperature effects on bacterial growth, colonization and community transition were reported on Kuytun 51 Glacier, where bacterial *Cyanobacteria* were dominant across the surface snow slope in the warming spring-summer, but rarely in the subsurface, winter-snow-layers (Xiang et al., 2009). As expected, the live cell density during the summer was high as a result of microbial growth in the surface snow. Other groups, Uetake et al. (2006), Yao et al. (2008), and Price and Bay (2012) also found that high microbial abundance was present in the warming spring-summer seasons in the Sofiyskiy glacier in the south Chuyskiy range of the Russian Altai, the Guoqu glacier in the Geladaindong mountain regions, and the deep Arctic and Antarctic ice cores, respectively. The obvious seasonal patterns of bacterial populations with a high cell density in summer strengthen the post-deposition effect on the microbial populations in glaciers.

In addition to those cell density peaks during the summer (open triangles in **Figures 2A,C**), there were also many density peaks in the spring from 1963 to 2000 (filled triangles in **Figures 2A,C**). The seasonal pattern of bacterial density was generally consistent with the dynamic mineral particle deposition with frequently dust outbreaks in spring and summer (**Figures 3** and **4** in this study; Wu et al., 2008; Liu et al., 2015). This indicated an important influence of dust deposition on the microbial communities in glaciers. All of the results suggest the fundamental contribution of dust-microbe deposition to the basic population pool size and the intensifying effect of post-deposition by microbial growth in the warming seasons.

### Geographic Difference of Microorganisms in the Glacier Ice

The present data showed that the *Polaromonas* sp. from the Dunde ice core clustered together more closely than those from other environments (**Figure 5**). The phenomena of *Polaromonas* sp. from the same location readily grouping together was also found in the Muztagata and Puruogangri glaciers (An et al., 2010; Xiang et al., 2010). Although *Polaromonas* sp. were widely distributed across the geographically different glaciers, statistical analyses demonstrated a large genetic distance among 43 unique glacier *Polaromonas* sequences, which positively associated with geographic distance (Franzetti et al., 2013). Similar geographic phenomenon of individual microorganisms was also found in the deep ice core. Bacteria *Alternaria* sp. were common in the deep ice cores of Greenland GISP2D and Antarctic Vostok 5G and Byrd, but their DNA sequences were phylogenetically different between the two polar regions (Knowlton et al., 2013). The geographic differences of *Polaromonas* sp. and *Alternaria* sp. across the isolated glaciers suggests that the mountain “barriers” to the microbial transportation can be surmounted by suitable adaptations, which leads to the geographic patterns of individual microorganisms.

Geographic differences are not only evident for *Polaromonas* sp. and *Alternaria* sp. but also for the taxonomic groups. It is obvious that there is a geographic distinction of taxonomic groups at the cryoconite habitats on three High-Arctic glaciers from the associated moraines and adjacent tundra on the Brøggerhalvøya peninsula, Svalbard (Edwards et al., 2013, 2014). Significant differences in the composition of dominant taxonomic groups are also found between alpine and Arctic cryoconite habitats (Edwards et al., 2014). The present data from the Dunde ice core showed that similar taxonomic groups frequently appeared along the ice core profiles as historical events (**Figures 8A1–A6**). Bacterial genus groups *Cryobacterium*-related, *Flavobacterium* sp., *Pedobacter* sp., *Polaromonas sp.*, and *Propionibacterium*/*Blastococcus* sp. were frequently found at the six tested ice layers of Dunde glacier from 1990 to 2000 (**Figures 8A1–A6**). Another example of similar group members sharing the related ice core layers can be found in the recently reported Dunde ice. Genera *Polaromonas* sp. and *Flavobacterium* sp. commonly found between 1990 to 2000 were also identified from the Dunde ice column AD 1780–1830 (Zhang et al., 2009). Although the dominant genus-related groups are similar in the related ice cores, the composition of the main genus-related groups is largely different across the geographically different glaciers. The bacteria *Cryobacterium*-related was more abundant in the Dunde ice cap than in the Muztagata glacier, while *Enterobacter* sp. appeared throughout the four tested ice layers of the Muztagata glacier but rarely in the Dunde ice cap (**Figures 8A1–A6,B1–B4**). Seven genus groups *Polaromonas* sp., *Enterobacter* sp., *Acinetobacter* sp., *Flexibacter* sp., *Thermus* sp., *Propionibacteria/Luteococcus* sp., and *Flavisolibacter* sp. were frequently identified in the four tested ice layers of Muztagata glacier from 1970–1988 (labeled as the dashed lines in **Figures 8B1–B4**), while *Polaromonas* sp. and *Flexibacter* sp. were found at all three tested ice columns of Puruogangri glacier from 1600 to 1920 (Zhang et al., 2009; An et al., 2010). All results clearly show that a few genus-related groups are dominant in the mountain ice cores and constitute the main taxonomic groups endemic to the local glacier regions. The difference of taxonomic group members across the geographically different glaciers suggests intermingling of the bacterial taxonomic groups to the point of geographic separation. More data of microorganisms in the deep ice are necessary for our better understanding of the biogeography of microorganisms in glaciers.

The geographic pattern of bacterial taxonomic groups could be attributed to the influence of the moisture and dust source area, which might vary across the mountain glaciers on the Tibetan Plateau (**Figure 1**; **Table 1**). Precipitation over the Muztagata glacier is mostly influenced by the westerly depressions, while precipitation over the Dunde ice cap and Puruogangri ice cap is mainly driven by the westerly depressions in winter and Indian monsoon in summer (Murakami, 1987; Wake et al., 1990; Davis et al., 2005). Dust in the mountain glacier Muztagata is mainly derived from deserts including Sary-Ishykotrau, Muyun Kum, Kyzyl Kum and Kara Kum, Taklimakan, and Gurbantunggut (**Figure 1**; Wake et al., 1993), while the Dunde ice cap is very close to the Gobi Desert, Qaidam Basin (**Figure 1**) and thus its dust components are more likely strongly affected by local dust storms and dominated by mineral particles from the two deserts Qaidam Basin and Tarim Taklimakan (Wu et al., 2009, 2010). The dramatic changes of the moisture sources and dust pathways across the mountainous glaciers may lead to differences in the microbial communities deposited in the glacier snow. Moreover, the heterogeneity of local conditions such as temperature, light intensity, melt-water availability, and nutrient concentrations in the snow may drive the spatial patterning of the microbial community by influencing the colonization of the dominant endemic species in the snow. Concerns on how surface communities are incorporated into the cores, how much they change after burial, and how the post processes contribute the geographic differences of microbial communities are still open questions. More data on the microbiological, meteorological, and physical and chemical characteristics of the glacier surface and subsurface snow and ice cores will be helpful for a better understanding of the biogeography of microorganisms in glaciers.

## Conclusion

The members of bacterial genus-related groups were found to be similar in the related ice cores at a historical scale but largely different between the two glaciers Muztagata and Dunde, even if microbial communities fluctuated along the two ice core depth profiles. Compared to the Muztagata glaciers, the Dunde ice core presented distinct members of the taxonomic groups. The five bacterial genus groups *Polaromonas*, *Pedobacter* sp., *Flavobacterium* sp., *Propionibacterium*/*Blastococcus* sp., and *Cryobacterium*-related frequently appeared at the six tested ice layers, constituting the dominant species endemic to the Dunde ice cap, while seven genus groups *Polaromonas* sp., *Enterobacter* sp., *Acinetobacter* sp., *Flexibacter* sp., *Thermus* sp., *Propionibacteria/Luteococcus* sp., and *Flavisolibacter* sp. were frequently found at the four tested ice depths of Muztagata glacier. The results demonstrate that the spatial differences in microbial communities between the two ice cores are more significant than the temporal differences. This study also showed a seasonal pattern of microbial cell density with high cell density occurring in the warming spring-summer.

## Author Contributions

YC: Design of the laboratory experiment outline, data collection, analysis, and interpretation, and draft of the manuscript. X-KL: Sequence data analysis, and interpretation. JS: Sequence data collection, analysis, and interpretation. G-JW: Mineral particle concentration examination of ice core, data analysis, and interpretation. L-DT: Oxgen isotope ratio analysis, and interpretation. S-RX: Design of the research outline, data analysis and interpretation, and revision of the manuscript.

## Conflict of Interest Statement

The authors declare that the research was conducted in the absence of any commercial or financial relationships that could be construed as a potential conflict of interest.

## Footnotes

1 http://decipher.cee.wisc.edu/FindChimerasOutputs.html

2 http://www.megasoftware.net/
